# Impacts of preweaning colostrum feeding practices and health measures on dairy cow production, while accounting for genetic potential

**DOI:** 10.1093/jas/skae061

**Published:** 2024-03-09

**Authors:** Elizah D McFarland, Ibrahim Elsohaby, Christine F Baes, Henrik Stryhn, Gregory Keefe, J T McClure

**Affiliations:** Department of Health Management, Atlantic Veterinary College, University of Prince Edward Island, Charlottetown, PE, Canada C1A 4P3; Department of Health Management, Atlantic Veterinary College, University of Prince Edward Island, Charlottetown, PE, Canada C1A 4P3; Department of Infectious Diseases and Public Health, Jockey Club College of Veterinary Medicine and Life Sciences, City University of Hong Kong, Hong Kong SAR; Centre of Genetic Improvement of Livestock, Department of Animal Biosciences, University of Guelph, ON, Canada N1G 2W1; Institute of Genetics, Vetsuisse Faculty, University of Bern, Bern 3012, Switzerland; Department of Health Management, Atlantic Veterinary College, University of Prince Edward Island, Charlottetown, PE, Canada C1A 4P3; Department of Health Management, Atlantic Veterinary College, University of Prince Edward Island, Charlottetown, PE, Canada C1A 4P3; Department of Health Management, Atlantic Veterinary College, University of Prince Edward Island, Charlottetown, PE, Canada C1A 4P3

**Keywords:** dairy calf management, genetics, milk yield, retrospective cohort

## Abstract

Calf management and health are essential for setting up the foundation of a productive cow. The objectives of this study were to estimate the impact of preweaning practices on milk production parameters while accounting for an animal’s genetic potential in New Brunswick, Canada. A retrospective cohort study was performed on 220 heifer calves from eight herds born in 2014-2015. Preweaning practices and health data were recorded by producers and reviewed by the herd veterinarian for each calf. The herd veterinarian also visited the farms to collect serum samples from calves and frozen colostrum samples. The production outcomes assessed were milk, protein and fat yields, standardized to 305 d for the first lactation (**L1**) and a combined group of lactations two and three (**L2 + 3**). The genomic potential was determined as genomic parent averages (**GPA**) for the associated production parameters. Analysis was performed with multivariable linear (L1) and linear mixed (L2 + 3) regression models. In L1, for every 1.0 kg increase in weaning weight, milk, protein, and fat yield increased by 25.5, 0.82, and 1.01 kg, respectively (*P *< 0.006). Colostrum feeding time (**CFT**) positively impacted L1 milk and protein production, with feeding between 1-2 h of life producing the greatest estimates of 626 kg of milk and 18.2 kg of protein yield (*P* < 0.007), compared to earlier or later CFT. Fat yield production was decreased by 80.5 kg (*P* < 0.006) in L1 when evaluating animals that developed a preweaning disease and were not treated with antibiotics compared to healthy untreated animals. Impacts on L2 + 3 were similar across all production outcomes, with a positive interaction effect of CFT and weaning weight. Compared to CFT < 1 h, the later CFT groups of 1-2 h and > 2 h produced greater yield outcomes of 68.2 to 72.6 kg for milk (*P* < 0.006), 2.06 to 2.15 kg for protein (*P *< 0.005), and 1.8 to 1.9 kg for fat (*P *< 0.045) for every 1 kg increase of weaning weight, respectively. The fit of all models was significantly improved with the inclusion of GPA. These results indicate that colostrum management and preweaning health measures impacted production parameters as adults. The inclusion of GPA significantly improved the accuracy of the models, indicating that this can be an important parameter to include in future studies.

## Teaser

A retrospective cohort analysis on how dairy calves are managed in the pre-weaning phase combined with their genetic potential could influence their adult production within three lactations. This study displayed positive and negative impacts of colostrum feeding time, weaning weight, disease occurrence, and antibiotic treatment on adult milk, protein, and fat yields.

## Introduction

In the last 100 yr, the dairy industry’s primary focus when assessing milk production outcomes has been management practices, nutrition, and genetics of mature and lactating cows, which has improved the quality of milk and dairy cow health and welfare (Miglior et al., 2017). Comparatively, studies on early life management practices affecting milk production have not been researched as extensively until recently. In the last century, heifer research’s primary focus has been growth and nutrition (Heinrichs et al., 2017; Uyama et al., 2022; Cavallini et al., 2023; Mellors et al., 2023). It was not until the last 20 yr the impacts of preweaning management practices were being evaluated on calf health, growth, and future productivity (Kertz et al., 2017). This change in focus could be related to the need to decrease mortality and morbidity rates and the availability of information from the increased collection of phenotypic and genetic information, allowing for earlier future production predictions for offspring.

In Canada, the mortality rate of preweaned calves is estimated to be 6% (Winder et al., 2018; Roche et al., 2020), which has not changed since 1986 (Waltner-Toews et al., 1986). Calf morbidity, while it has been described regarding specific diseases or in association with antibiotics in smaller regions (Windeyer et al., 2014; Uyama et al., 2022), has not yet been described overall across Canada (Winder et al., 2018); therefore, the closest comparison would be to the United States. In a 2014 national survey in the United States, morbidity was reported to be 33.8%, and the most common diseases affecting preweaned heifers were calf diarrhea (50.9%) and respiratory disease (28.1%) (Urie et al., 2018; NAHMS, 2021a). Of the calves surveyed, 12% had developed failure of transfer of passive immunity (**FTPI**) (serum immunoglobulin G (**IgG**) concentration < 10g/L), which could be an indication of improper colostrum management (Lombard et al., 2020). A large component of preweaning calf research has been dedicated to understanding the influences of FTPI, which are significantly impacted by various colostrum management factors, from the time at feeding after birth, quality of colostrum to the volume of colostrum fed (Weaver et al., 2000; Godden et al., 2019). While there has been a greater emphasis on calf health and management practices (Winder et al., 2018; Elsohaby et al., 2019; Abuelo et al., 2021), there are few studies that investigate the impacts past the first lactation and on multiple herds (Faber et al., 2005; Heinrichs and Heinrichs, 2011; Soberon et al., 2012). Investigating these later impacts can be imperative as the future success of cows starts as calves. In a study by Volkmann et al. (2019), they note that after the first lactation, health and fertility are critical for long, efficient lives, which can be grounded in preweaning rearing.

In addition to different management practices, such as colostrum management and treatment protocols, an animal’s genetic disposition can also play a role in the preweaning period, altering the phenotypic expression of milk yield (Soberon et al., 2012). Over the last two decades, the implementation of conventional genetic selection has played a significant role in advancing milk production through early predictions (Miglior et al., 2017; Brito et al., 2021). In the case of non-production based traits, such as health and longevity, the environment an animal is in has a large influence on its phenotypic expression. As a result of this, breeding selection programs include available genomic information for more accurate estimations of all traits (Bengtsson et al., 2020). Even with this knowledge, it has only been recently that Canada has started to investigate the inclusion of calf health into selection programs (van Staaveren et al., 2023). Accounting for the calf’s genetics or genetic potential could improve the estimation of calfhood management practices and calfhood diseases’ impact on the animal’s production potential once they enter the lactating herd. To the authors’ knowledge, no studies have considered all three components of calf health, management, and genetic potential and their impact on production together.

The objective of this study was to estimate the impact that colostrum feeding practices and health measures during a calf’s preweaning period have on future production performance (milk, protein, and fat yields) in first and subsequent lactations, while accounting for the animal’s genetic potential, in New Brunswick, Canada.

## Materials and Methods

### Data Sets

A retrospective cohort study was conducted using two pre-existing datasets from 2014 to 2021. All animals and procedures used were approved by the Animal Care Committee of the University of Prince Edward Island (#19-014).

The first dataset was repurposed from previous provincial research and contained information on preweaning calf management practices and diseases. Herd participation was derived from the 24 genotyped herds included in the New Brunswick Dairy Genome Project (**NBDGP**), who agreed to have additional information collected. A total of 470 Holstein heifer calves, born in 2014-2015, were enrolled from eight herds in New Brunswick, Canada. Producers recorded data and were instructed to follow their own current operation’s on-farm calf management practices. They were provided with a study recording sheet and instructed to create a log for each calf born during the study period and collect colostrum samples. The participating herd veterinarian further reviewed all data recorded.

All herds recorded the following information in each calf log; colostrum practices regarding the first feeding of colostrum, consisting of colostrum feeding time (**CFT**) (< 1 h, 1-2 h, > 2 h), volume consumed (range: 1-5 L, dichotomized to ≤2 L and >2 L due to distribution), method of feeding (assisted feeding via bottle or tube feeding, pail, and other), colostrum type (fresh from the dam, pooled, replacer), and colostrum preservation (fresh, frozen, pasteurized, acidified, refrigerated); preweaning disease occurrence (diseases recorded were calf diarrhea, respiratory disease, and navel infection, which were merged into one binary trait if an event of preweaning disease occurred due to number of records) and birth weight and weaning weights (**WWT**) (a digital scale was provided for each farm). If producers identified an incidence of disease, it was confirmed by the herd veterinarian. If antibiotic treatment was required, it was prescribed by the herd veterinarian (both case-based and prophylactically) and recorded as a binary trait (yes, no). Records were only reported during the preweaning phase, with calves’ age at weaning at the producer’s discretion, and ranged from 43 to 104 d. Colostrum samples from the first feeding were collected by producers and frozen until picked up. The veterinarian collected blood and spun serum samples on calves between 2 and 18 d of age at their earliest convenience. Blood serum and colostrum samples were sent to researchers to assess the quality and passive immunity status using infrared spectrometry. Hair root samples (collected between 1 d of age and a month before first calving) were collected for genotyping. Data were evaluated to determine potential categorical groupings for CFT and colostrum volume fed and were chosen based on the natural distribution of the data.

The second dataset focused on phenotypic production characteristics of the NBDGP enrolled animals and consisted of milk, fat, and protein yields (kg), standardized to a 305-d lactation. Phenotypic lactation curves (305 d) were obtained from DHIA databases, which were estimated with multiple trait prediction models, previously explained by Schaeffer and Jamrozik (1996). Records were obtained up to an animal’s third lactation. An animal’s genetic potential was determined by its genomic parent average (**GPA**). The GPA was estimated with an animal’s parents’ genomic estimated breeding values (**GEBV**) from national evaluations using the following equation:


$$\begin{array}{l} GPAanimal=\frac{\left(GEBVsire+GEBVdam\right)}{2} \end{array}$$

**Figure 1.**
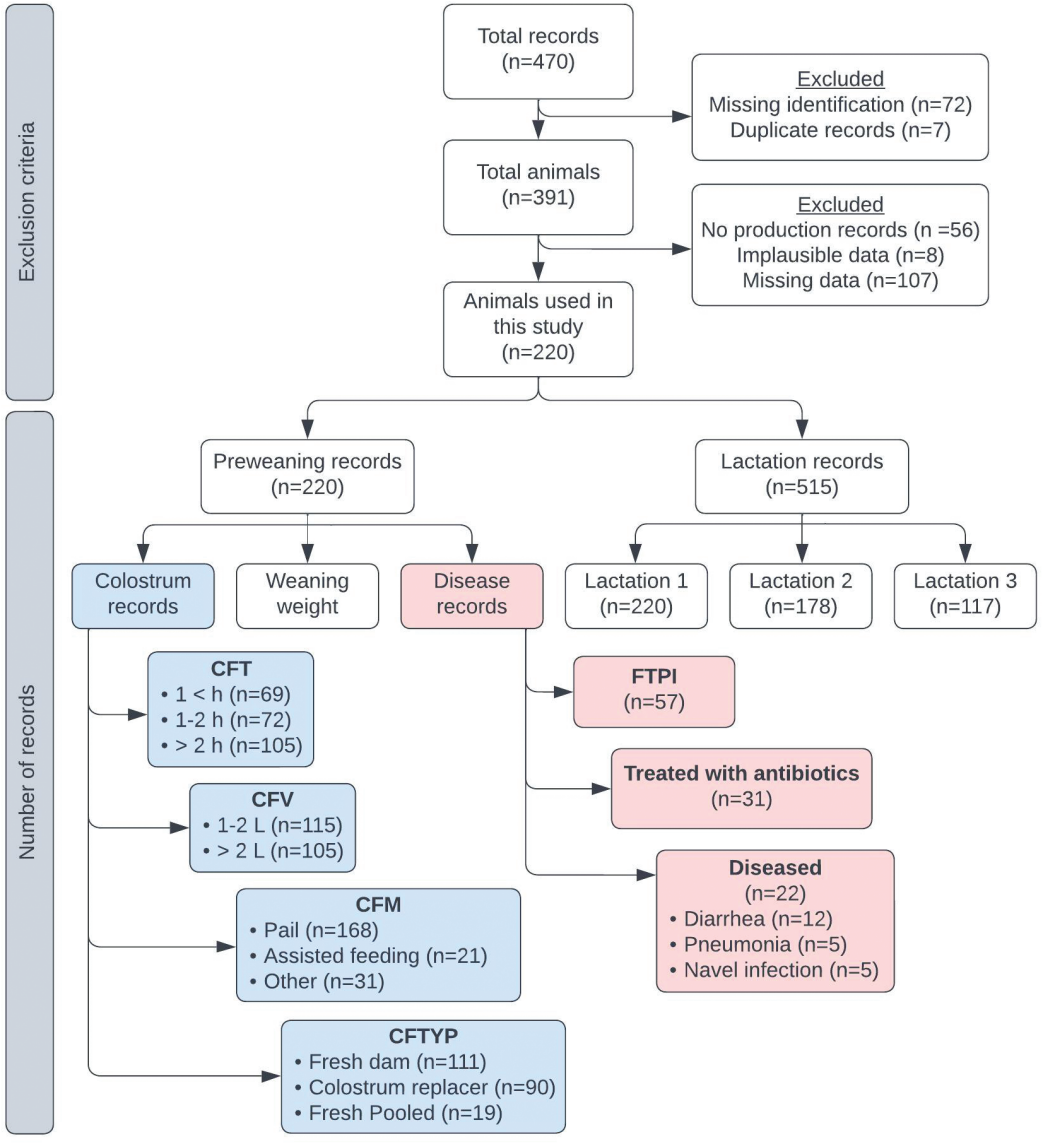
Flowchart showing the exclusion criteria for records used and the final breakdown of the number of records for each variable. CFT = time to first feeding of colostrum after birth; CFV = volume of colostrum fed at first feeding; CFM = feeding method for first colostrum feeding; CFTYP = type of colostrum fed at first colostrum feeding; FTPI = failure of transfer of passive immunity.

Data sets were merged and matched to each calf record using their Lactanet (Sainte-Anne-de-Bellevue, QC, Canada) and Holstein Canada (Brantford, ON, Canada) identification numbers. The dataset was cleaned and edited for analysis. The final dataset had 220 calves from the NBDGP study with complete records on the preweaning management practices and production information for at least one lactation (exclusion criterion displayed in Fig. 1). The number of participating calves across herds ranged from 6 to 67, averaging 28 per herd. The number of cows per lactation included, L1 = 220, L2 = 178, and L3 = 117. Lactations were split into two groups for analysis, where lactation one (**L1**) was analyzed independently from lactations two and three (**L2 + 3**).

### Statistical Analysis

Six models were developed, one for each of the three production outcomes (cumulative milk, fat and protein yield (kg) standardized to 305 d) across the two lactation groups of L1 and L2 + 3, where each observation was an individual lactation record. For the L1 records, analysis was conducted on the three outcomes with linear multivariable models. The L2 + L3 records contained repeated measures (one observation per lactation), and analysis was conducted on the three outcomes with linear mixed multivariable models using a two-level hierarchical structure. Seventeen cow-level predictor variables were initially assessed by univariable linear regression or mixed linear regression analysis for the L1 and L2 + 3 models, respectively, and are listed in Table 1.

**Table 1.** Estimated means and correlation coefficients (*r*) of predictors from univariable analysis for the outcomes of milk, protein, and fat yields standardized to a 305d lactation in each lactation group analyzed (L1 = lactation 1, L2 + 3 = lactation 2 and 3)

I—Categorical variables^1^	N	Milk yield, kg				Protein yield, kg				Fat yield, kg			
		L1		L2 + 3		L1		L2 + 3		L1		L2 + 3	
		Mean	*P*-value	Mean	*P*-value	Mean	*P*-value	Mean	*P*-value	Mean	*P*-value	Mean	*P*-value
First colostrum feeding time^2^													
< 1 h	69	8,488.6	<0.001	11,627.1	0.046	268.8	<0.001	366.8	<0.001	336.3	<0.001	449.2	<0.001
1-2 h	72	9,157.2		11,731.2		290.3		373.0		352.8		456.3	
> 2 h	79	9,839.0		12,334.0		318.7		400.3		386.2		502.3	
First colostrum feeding volume^2^													
≤ 2 L	115	8,657.8	<0.001	11,488.0	<0.001	275.8	<0.001	365.5	<0.001	336.0	<0.001	451.4	<0.001
> 2 L	105	9,777.8		12,393.6		313.5		398.0		385.5		491.4	
First colostrum feeding type^2^													
Fresh	111	9,889.7	<0.001	12,355.1	<0.001	315.0	<0.001	394.1	<0.001	388.2	<0.001	491.7	<0.001
Replacer	90	8,378.8		11,349.3		268.4		362.9		327.3		443.7	
Pooled	19	8,971.6		11,545.9		289.8		375.1		345.6		445.5	
Navel disease													
Yes	5	9,129.8	0.948	13,105.5	0.112	297.8	0.895	418.4	0.117	378.4	0.627	528.7	0.081
No	215	9,193.8		11,872.0		293.7		379.4		359.2		468.2	
Calf diarrhea													
Yes	12	8,463.5	0.233	10,467.3	0.005	276.6	0.376	345.3	0.031	332.0	0.260	424.7	0.047
No	208	9,234.4		11,993.4		294.7		382.6		361.2		472.6	
Respiratory disease													
Yes	5	7,942.0	0.194	13,111.9	0.112	246.8	0.123	405.5	0.301	312.8	0.226	494.9	0.459
No	215	9,221.4		11,871.8		294.8		379.8		360.7		469.2	
Preweaning disease occurrence													
Yes	22	8,496.4	0.113	11,748.0	0.658	274.6	0.171	377.6	0.804	338.2	0.225	466.9	0.855
No	198	9,269.7		11,925.3		295.9		380.8		362.0		470.2	
Antibiotic treatment													
Yes	31	9,147.9	0.902	12,421.6	0.110	290.2	0.755	393.5	0.208	358.6	0.945	489.8	0.167
No	189	9,199.6		11,826.9		294.3		378.5		359.8		466.8	
FTPI ^3^													
Yes	59	8,549.6	0.009	11,413.2	0.017	273.9	0.011	365.6	0.024	332.3	0.006	449.2	0.025
No	161	9,417.1		12,088.7		300.7		386.0		369.2		477.5	
II—Continuous variables	Mean	*r*	*P*-value	*r*	*P*-value	*r*	*P*-value	*r*	*P*-value	*r*	*P*-value	*r*	*P*-value
Birth weight, kg	41.3	0.314	<0.001	0.265	<0.001	0.321	<0.001	0.325	<0.001	0.3291	<0.001	0.326	<0.001
Weaning weight, kg	84.9	0.215	<0.001	0.190	<0.001	0.231	<0.001	0.216	<0.001	0.2492	<0.001	0.162	<0.001
Average daily gain, kg/day^4^	0.008	0.066	0.329	0.026	0.563	0.060	0.379	0.066	0.261	0.0624	0.355	0.128	0.028
Days to weaning, d	60.6	0.028	0.668	0.041	0.448	0.045	0.512	0.032	0.586	0.0224	0.752	0.072	0.219
GPA^5^ milk	44.3	0.156	0.020	0.124	<0.001	—	—	—		—	—	—	—
GPA protein	1.9	—	—	—	—	0.109	0.109	0.105	<0.001	—	—	—	—
GPA fat	2.7	—	—	—	—	—	—	—		0.0346	0.612	0.124	0.004

The significance for univariable association was deemed at *P *< 0.2 for further analysis.

^1^Herd was included in the model as a fixed effect. However, for simplicity, herd estimates were removed from the table to focus on the predictors of interest.

^2^First colostrum feeding management factors.

^3^FTPI = failure of transfer of passive immunity, with immunoglobulin cut-off of 10 g/L.

^4^Average daily gain is calculated by: (weaning weight—birth weight)/number of days between birth and weaning.

^5^GPA = genomic parent average for the associated outcome.

Predictor variables with *P *< 0.2 or those identified as confounders were retained for the multivariable analyses, which used backward stepwise regression with a significance of *P *≤ 0.05. The primary exposures were focused on colostrum practices at birth and health-related events. Herd was identified as a confounder and forced in the models as a fixed effect. Potential interactions were identified from causal reasoning (using causal diagrams) and their biological relevance. Statistical significance was derived from Wald tests, and categorical predictors were further by pairwise comparisons. Interactions were also visualized with interaction plots. Model assumptions were assessed graphically using the standardized residuals.

The final merged data were analyzed using R Studio (RStudio Team, Boston, MA) and Stata software (version 17, StataCorp, College Station, TX). See Supplementary Material for complete symbolic notation of the final models.

## Results

### Descriptive Statistics

Descriptive statistics of calf management variables and outcomes across the 220 calves and univariable analysis are outlined in Table 1. The following predictors were retained for multivariable analysis; categorical predictor variables; CFT, < 1 h, 1-2 h, and > 2 h, with 31.3%, 32.7%, and 35.9% of calves in each group, respectively; preweaning disease occurrence was reported in 22 calves (10.0%), which was attributed to calf diarrhea (*n* = 12), respiratory disease (*n* = 5), and navel infection (*n* = 5); and antimicrobial treatment given to 31 (14.1%) calves; continuous predictor variables included; WWT (mean = 84.9 kg (SE: 0.77 kg)) and the associated GPAs for each production outcome, with ranges of −952 to 1381 kg for milk yield, −65 to 63.5 kg for fat yield, and −40.5 to 41 kg for protein yield.

Adult production values for 305 d milk, protein and fat yield were normally distributed for L1 and L2 + 3. The means for each outcome for L1 were: milk 9192.3 kg (SE: 146.5 kg), protein 293.7 kg (SE: 4.6 kg), and fat 359.6 kg (SE: 5.88 kg). The means for each outcome for L2 + 3 were: milk 11,905.5 kg (SE: 2,161.6 kg), protein 380.5 kg (SE: 69.3 kg), and fat 469.8 kg (SE: 96.7 kg).

### Accounting for Genetic Potential

To account for genetic potential, GPA was included in all models and was significant in all six models (*P* < 0.001). The estimates produced for GPA were small, stating that for every one-unit increase in GPA, the corresponding production yield would increase within the range of 0.90 to 1.2 kg per lactation. However, the inclusion of GPA significantly improved the fit of all models. The *R*^2^ for models without and with GPA for L1 production outcomes increased as follows: milk yield *R*^2^ increased from 0.58 to 0.63, fat yield *R*^2^ increased from 0.64 to 0.69, and protein yield *R*^2^ increased from 0.65 to 0.68. For L2 + 3 mixed models, model fit was accessed by the difference in log-likelihood ratios. The increase in log-likelihood ratios between models without and with GPA was 6.75 for milk yield, 16.12 for fat yield, and 8.84 for protein yield.

### Analytical Statistics

Table 1 shows the *P*-values associated with univariable analysis for animals in L1 and L2 + 3. Of eighteen possible predictor variables, 11, 10, and 7 univariable associations were identified for L1 multivariable modelling for milk, protein, and fat yield, respectively. Ten, 11, and 9 univariable associations were identified for L2 + 3 multivariable modelling for milk, protein, and fat yield, respectively.

*Lactation 1 models*. Colostrum feeding time and WWT were the only predictive variables retained in the L1 multivariable linear models for milk yield and protein yield (Table 2). For the L1 milk yield model, calves whose colostrum was fed between 1 and 2 h produced 625 kg more milk than those whose colostrum was fed < 1 h (*P *= 0.007) and 468 kg more than those whose colostrum was fed > 2 h (*P *= 0.053). A similar trend was observed with protein yield, where calves whose colostrum was fed between 1 and 2 h produced 18 kg more protein than those whose colostrum was fed < 1 h (*P *= 0.007) and 12 kg more than those whose colostrum was fed > 2 h (*P *= 0.083). Greater WWT positively affected milk and protein production with an increase of 25.5 kg (SE: 9.3 kg) in milk yield and 0.82 (SE: 0.27 kg) in protein yield for every one kg increase in WWT. Therefore, a cow with the median WWT in our study of 84.5 kg would produce 165.7 kg of milk yield and 5.3 kg of protein yield more in the first lactation compared to calves weighing in the 25th percentile of 78 kg at weaning.

**Table 2.** Estimate summary of lactation one linear models, where the outcome for each model was milk, protein, and fat yield, respectively, with the values of each outcome standardized to a 305-d lactation (kg)

Variable^1^	Milk yield, kg			Protein yield, kg			Fat yield, kg		
	Estimate	SE	*P*-value	Estimate	SE	*P*-value	Estimate	SE	*P*-value
Colostrum feeding time									
< 1 h	8,930.8	169.0		285.6	5.0			-	
1-2 h	9,556.7	160.7	0.017	303.9	4.7	0.022		-	
> 2 h	9,088.7	165.6		291.6	4.8			-	
Weaning weight, kg	25.5	9.3	0.006	0.82	0.3	0.003	1.0	0.4	0.004
D × TX^2^									
Healthy and no antibiotics		-			-		362.5	3.7	0.041
Healthy and antibiotics received		-			-		345.2	15.0	
Diseased and no antibiotics		-			-		282.0	28.3	
Diseased and antibiotics received		-			-		357.7	12.1	
GPA^3^	1.0	0.2	<0.001	0.9	0.2	<0.001	0.9	0.2	<0.001

The estimated values are the differences in yield (kg) of a lactation one cow who received the corresponding management practice as a calf. There were 220 observations for each model.

^1^Herd was included in the model as a fixed effect. However, for simplicity, herd estimates were removed from the table to focus on the predictors of interest.

^2^Interaction between preweaning disease occurrence (D) and antibiotic treatment (TX). The main effects are not presented as they are a part of the interaction and the *P*-value presented is for the overall main effects and interaction term.

^3^GPA: genomic parent average for the associated production yield outcome.

**Figure 2.**
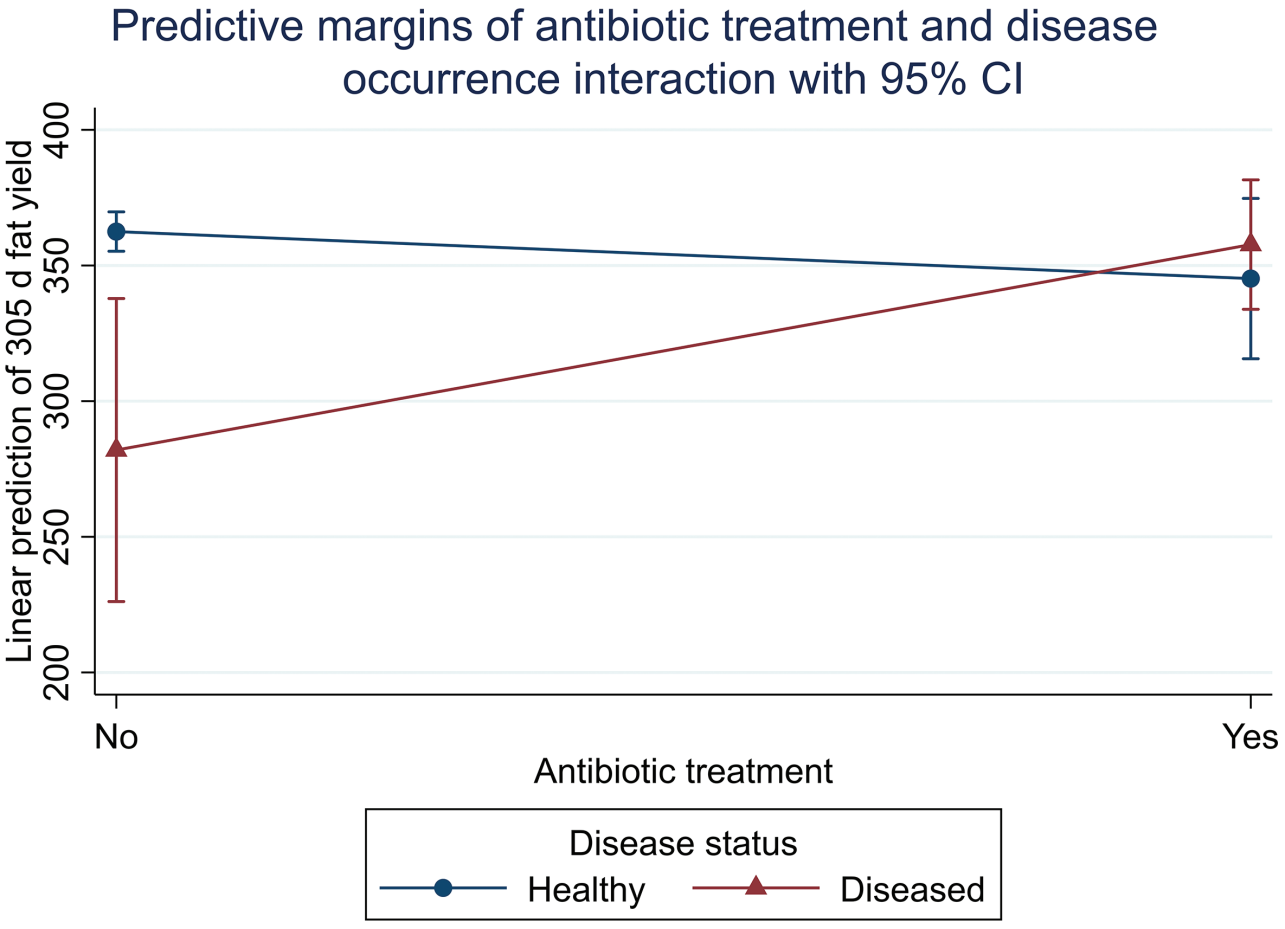
The interaction effect of preweaning antibiotic treatment (no, yes) and disease occurrence (healthy, disease) on predicted lactation 1 for 305 d fat yield. The *y*-axis displays the total predicted yield in lactation 1.

The predictor variables retained in the L1 multivariable linear model for fat yield were WWT, preweaning disease occurrence, and antimicrobial treatment (Table 2). Like milk and protein yield, greater WWT positively affected fat yield with an increase of 1.01 kg (SE: 0.35 kg) for every 1 kg increase in WWT. Therefore, a cow with the median WWT in our study of 84.5 kg would produce 6.6 kg more milk fat in the first lactation compared to calves weighing the 25th percentile of 78 kg at weaning. An animal’s disease status and potential antibiotic treatment negatively affected fat yield, and there was a significant disease-treatment interaction (*P* = 0.007), indicating that the interpretation of these two variables depends on the level of the other variable. Compared to animals that were healthy and untreated with antibiotics in the preweaning period, calves who developed a disease and left untreated with antibiotics produced 80.5 kg less fat, animals that were healthy and treated with antimicrobials produced 17.3 kg less, and animals that were both diseased and treated with antibiotics had a produced 4.7 kg less fat. Figure 2 shows this interaction, whereby the untreated sick calf had the lowest fat yield.

**Figure 3.**
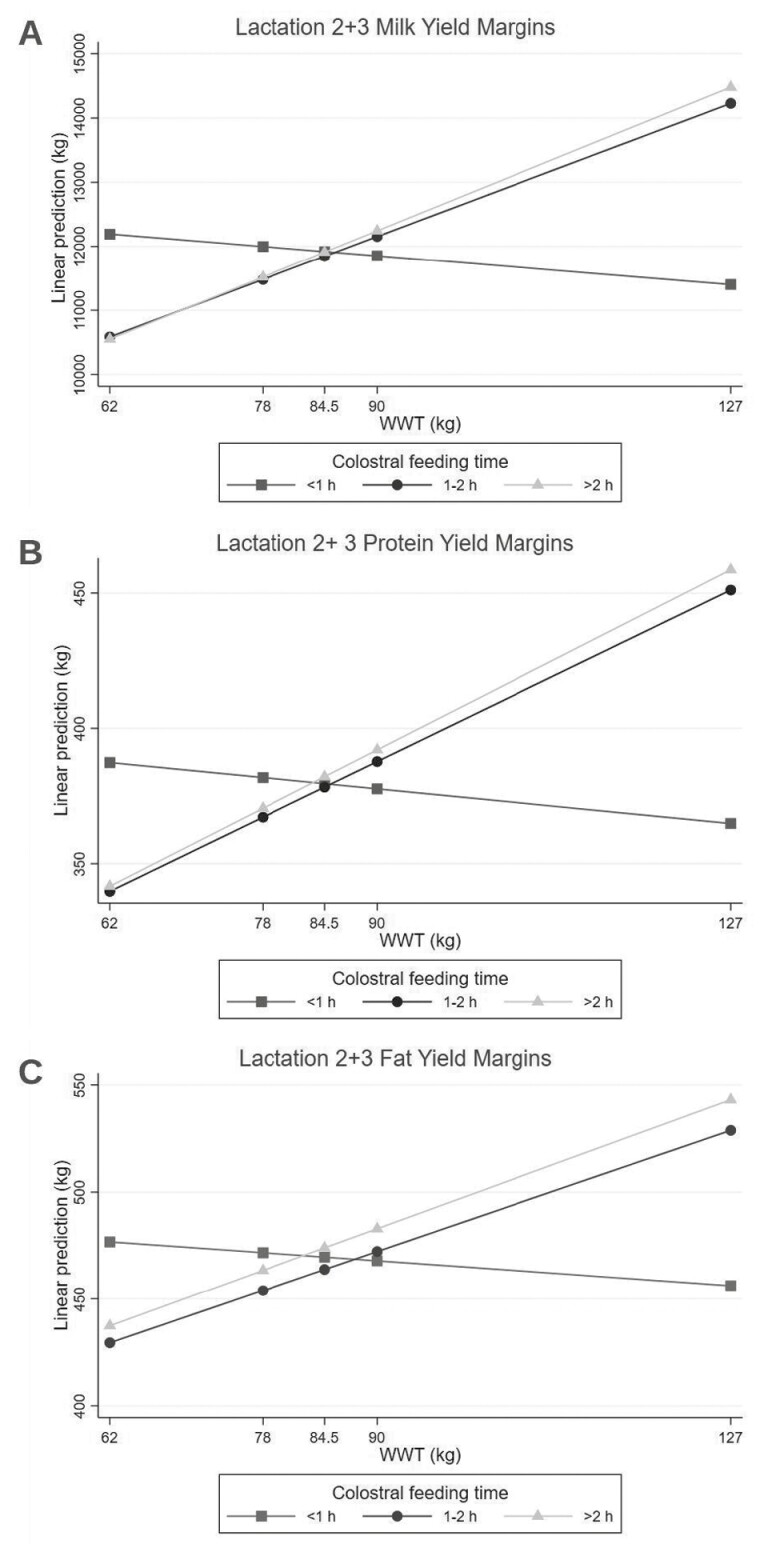
The interaction effect of colostral feeding time and weaning weight (WWT) on predicted lactation 2 + 3, (a) milk, (b) protein, and (c) fat yields. The *y*-axis displays the total predicted yield in lactation 2 + 3 (based off the model constant).

*Lactation 2 + 3 models.* The L2 + 3 linear mixed models for milk yield and protein yield resulted in CFT and WWT being predictive variables, with an interaction between the two variables (Table 3). Again, the interpretation of the interaction variables is dependent on the other variable’s level in the interaction. Therefore, interpreting the coefficients in Table 3 in isolation is misleading; however, the combined effects of the coefficients are shown in Fig. 3a-c and described below. Calves with CFT of < 1 h were used as the referent group and displayed a decline of 12 kg in milk yield per 1 kg increase of WWT. The CFT groups of 1-2 h and > 2 h had significant increases in milk yield of 56 and 60 kg per 1 kg increase of WWT, respectively (Fig. 3a). Protein yield also displayed similar results to milk yield for the CFT and WWT interaction, where CFT groups of 1-2 and > 2 h had significant increases of protein yield by 1.71 and 1.80 kg per 1 kg increase of WWT, respectively, compared to CFT < 1 h (Fig. 3b). The three lines cross at approximately 84.5 kg of WWT (the median WWT); therefore, regardless of CFT, calves with a WWT of 84.5 kg produced on average approximately 12,000 kg of milk (Fig. 3a) and 380 kg of protein (Fig. 3b) in L2 + 3. Conversely, for calves at the 75th percentile for WWT (90 kg), the cows produced on average approximately 346.6 kg of milk (Fig. 3a) and 12.2 kg of protein (Fig. 3b) more in L2 + 3 when CFT was 1-2 h and > 2 h compared to when CFT was < 1 h.

**Table 3.** Estimates summary of lactation two and three linear mixed models, where the outcome for each model was milk, protein, and fat yield, respectively, with the values of each outcome standardized to a 305-d lactation (kg)

Variable^1^	Milk yield, kg			Protein yield, kg			Fat yield, kg		
	Estimate	SE	*P*-value	Estimate	SE	*P*- value	Estimate	SE	*P*-value
Colostrum feeding time^2^									
< 1 h	Referent			Referent			Referent		
1-2 h	−73.1	278.3		−1.2	8.2		−8.5	11.3	
> 2 h	−8.2	309.3		2.6	9.1		1.0	12.5	
Colostrum feeding time × Weaning weight^3^									
<1 h	−12.2	18.2	0.503	−0.4	0.5	0.515	−0.3	0.7	0.640
1-2 h	56.0	18.1	0.002	1.7	0.5	<0.001	1.5	0.7	0.024
>2 h	60.4	19.3	0.002	1.8	0.6	<0.001	1.6	0.7	0.022
GPA	0.92	0.24	<0.001	1.0	0.2	<0.001	1.2	0.2	<0.001
Parity	280.9	145.4	0.053	9.7	4.6	0.036	10.9	6.2	0.077

The estimate values are the differences in yield (kg) in lactation two and three cows who received the corresponding management practice as a calf. There were 220 animals, with a total of 295 observations for each model.

^1^Herd was included in the model as a fixed effect. However, for simplicity, herd estimates were removed from the table to focus on the predictors of interest.

^2^Estimates presented are at the weaning weight intercept, which was centred at 84.5 kg (median).

^3^Interaction between colostrum feeding time and weaning weight, *P*-values with degrees of freedom = 2.

^4^GPA = genomic parent average for the associated production yield outcome.

For fat yield in L2 + 3, a model including an interaction with preweaning disease occurrence and antibiotic treatment was developed to compare to the L1 fat yield model; however, those variables were not significant. The final mixed model resulted in CFT and WWT being the predictive variables and as an interaction variable (CFT × WWT), similar to the L2 + 3 milk and protein yield models. Similar directions for the impact of CFT and WWT were observed. The interaction term, CFT × WWT, indicated that CFT of 1-2 h and > 2 h had increased fat yield of 1.53 to 1.63 kg, respectively, for every 1.0 kg increase of WWT (Fig. 3c). The three lines cross at approximately 84.5 kg of WWT (the median WWT); therefore, regardless of CFT, calves with a WWT of 84.5 kg produced on average approximately 454 kg of at (Fig. 3c) in L2 + 3. Conversely, for calves at the 75th percentile for WWT (90 kg), the cows produced on average approximately 9.7 kg of fat (Fig. 3c) more in L2 + 3 when CFT was 1-2 h and > 2 h compared to when CFT was < 1 h.

## Discussion

To our knowledge, the present study is the first to estimate the impact of preweaning colostrum management and measures of health that could influence a dairy heifer’s future production performance while accounting for their genetic potential. We hypothesized that including an animal’s genetic potential would allow for an analysis of how environmental impacts could influence the predicted capability of an animal and improve the estimate of the impact of colostrum management practices, calfhood disease, and WWT in preweaned calves on their future lactations. Genetic improvement and analysis are important for the livestock industry (Faber et al., 2005). We used GPA to estimate an animal’s genomic potential because it is important to know an offspring’s milk production ability to assess the impact of preweaned calf factors on future productivity more appropriately. Using GPA improved the fit of the models by 5%-8% for L1 and 6.75-16.12 units for L2 + 3 using *R*^2^ (L1) and L.R. (L2 + 3). The estimated effects of independent variables also differed between models with and without GPA included (data not included). This supports that GPA is important and improved models assessing preweaning variables on production outcomes. In a study by Faber et al. (2005), sire and dam parental transmitting abilities were used to account for genetics in a model evaluating the effects of colostrum ingestion in future lactations; by evaluating genetics with parental information, the outcomes focused on the genetic superiority of the parents. Compared to our study, the focus was on what an individual animal (and their genetic potential) is capable of. However, the authors laid the groundwork for incorporating genetics with calf management factors in an epidemiological approach. Faber et al. (2005) and our study showed that genetics should be applied in modelling calf management effects, particularly colostrum feeding practices, to improve variable estimates and models.

Colostrum feeding time positively impacted L1 adult cow production, with CFT of 1 to 2 h having the most significant milk and protein production increase in a 305-d lactation. The significant difference between CFT of 1 to 2 h compared to earlier and later times is an interesting finding. Most literature and management standards recommend feeding colostrum as early as possible or within the first two hours of life (Lora et al., 2018; NAHMS, 2021b). A potential explanation for this is that (1) calves fed in the first hour of life may absorb colostrum less efficiently due to slower gut motility due to the stress of parturition (Stott et al., 1976; Fischer et al., 2018; Smith et al., 2022), (2) calves fed past two hours of birth may have less efficient absorption of colostrum IgG due to progressive gut closure (Osaka et al., 2014), and (3) the confounding effect of herd. The reason that the < 1 h CFT group displayed decreased production cannot be determined by this observational study, but we can hypothesize potential causes. The general immunity of the calf and gut closure plays a vital role within the first 24 h of life. The Canadian Code of Practice for Dairy Cattle (National Farm Animal Care Council, 2023) requires calves to be fed no later than 6 h after birth, as this is a point where IgG absorption declines. A study by Fischer et al. (2018) went further and suggested that there may be a more critical time point between 1 and 6 h of life when the closure of the small intestine progresses to a degree. Other hypotheses include the gut microbial community and hormonal influences within the first hour of life (Kruse and Buus, 1972; Malmuthuge et al., 2015; Fischer et al., 2018). Literature has reported a variety of microbes that begin colonizing within the first 24 h from beneficial bacteria, such as *Bifidobacterium* and *Lactobacillus*, and potentially harmful gut microbes, such as *Escherichia coli* (Lanz Uhde et al., 2008; Malmuthuge et al., 2015). Current reports of the distribution of pathogens tested at different CFTs are conflicting. As calves fed within the first 45 min of life have shown increased concentration of IgG and rapid colonization of *Bifidobacterium* and *E. coli* (Fischer et al., 2018), compared to later feeding times have decreased microbes and shifted establishment of different microbial dynamics in the gut (Malmuthuge et al., 2015; Fischer et al., 2018). Others have reported that absorption of IgG was decreased with increased bacterial presence, leaving the animal at greater risk of developing FTPI, which has associations with decreased adult production (Johnson et al., 2007; Elsohaby et al., 2019; Smith et al., 2022). Hormonal changes in the early hours of life due to increased stress from birth or delayed colostrum feeding have been hypothesized to cause cortisol shock, which changes the absorptive capacity of the intestine (Kruse and Buus, 1972; Fischer et al., 2018). However, this has equally been disputed by other studies that have reported no relationship between cortisol concentrations and IgG absorption (Stott and Reinhard, 1978). While our study did not look at the gut microbiota, bacterial counts of the colostrum, or hormonal impacts, our data suggest these could be possible effects that have resulted in CFT 1 to 2 h calves outperforming the CFT < 1 h and should be investigated further in future studies designed to study this issue prospectively. Next, CFT of 1 to 2 h also outperformed CFT > 2 h in L1, which could be associated with reduced colostrum IgG absorption as the calf ages. The transfer of immunoglobulins across the calf’s gut epithelium progressively declines until gut closure at 18 to 24 h of age (Lora et al., 2018; Godden et al., 2019). Therefore, the longer a calf goes without receiving colostrum, the greater chance that the calf has FTPI, making it more susceptible to calfhood diseases, which may impact its future lactation performance (Stott et al., 1979; Michanek et al., 1989; NAHMS, 2012, 2021b; Godden et al., 2019). In the present study, most calves were first fed colostrum within the Canadian guidelines < 6 h of life (National Farm Animal Care Council, 2023). However, in this study, producers who could feed within 1-2 h of life had increased milk and protein yields for the first lactation. This effect is also potentially due to the confounding effect of herd, seen in the univariate and final model estimates. In Table 1, the univariate estimates of CFT without herd included did not display this pattern but show that production increases linearly from < 1 to > 2 h. Once CFT was included in the final model with herd, the effect of improved milk and protein production in the first lactation from feeding colostrum at 1 to 2 h was displayed compared to other CFT groups. Due to this relationship herd remained in the model to account for the confounding present. While confounding was present, the effect was observed when looking at the mean production values across CFT and herds. It would benefit future studies to explore this potential relationship further.

Of all colostrum factors evaluated, CFT was the only significant colostrum management practice for both L1 and L2 + 3 final models. Different components related to colostrum feeding practices are known to be closely related to each other and may impact each other in an analysis. Specifically, the differences between the CFT groups may also be a reflection of the volume of colostrum fed (Faber et al., 2005; Lora et al., 2018; Winder et al., 2018) and quality of colostrum and amount of IgG fed to calves (Godden et al., 2019; Armengol and Fraile, 2020; Elsohaby et al., 2020). In view of this, evaluating CFT on its own can have some implications due to how interconnected it is to other practices. A previous study found the risk of a calf developing FTPI increases by 13% with every hour delayed to first colostrum feeding, however, the risk decreases by 59% and 3%, respectively, with every additional liter of colostrum given and every additional gram of IgG/L in the colostrum fed (Lora et al., 2018). Consequently, while feeding a calf as early as possible (<2 h) is a key component, it does not guarantee they are receiving enough IgG to ensure sufficient passive immunity. Colostrum quality was assessed in this study; however, 30% of samples were missing results or had unreliable values, thus this variable had to be removed from model building. Of the colostrum samples with IgG concentration results (*n* = 154, range: 0.62 to 265.73 g/L), only 58% of calves were fed colostrum above the recommended 50 g IgG/L. Across the categories of CFT a majority of calves were fed adequate amounts of IgG above 50 g/L, <1 h = 65%, 1 to 2 h 52%, and > 2 h = 60%, so it is possible that those consuming adequate IgG is not as affected by the CFT (Lora et al., 2018; Geiger, 2020). The relationship was explored further by assessing the distribution of CFT from the first feeding against the available information from the second feeding. For this study, the information on second colostrum feeding was limited, because while most calves across participating herds received a second feeding of colostrum, the information was not consistently recorded across variables (time, volume, method). The second CFT was the most complete variable (*n* = 211) and was assessed against the first CFT. For the first CFT of < 1 h (*n* = 63), the range of second colostrum feeding ranged from 3 to 24 h, with a mean of 10.5 h. First CFT of 1 to 2 h (*n* = 72), the range of second colostrum feeding ranged from 1 to 19 h, with a mean of 11.3 h. First CFT > 2 h (*n* = 76), the range of second colostrum feeding ranged from 2 to 26 h, with a mean of 13.9 h. From this information, the time a calf received a second feeding of colostrum was distributed evenly across the first CFT group and unlikely a confounder in this study. In the final analysis, while many variables related to colostrum feeding practices were recorded in the current study, CFT was the only significant variable in univariable and final analysis. This could be because CFT correlates with some of these other colostrum variables, in turn masking their significance in our models, which should be considered upon interpretation.

Deciding to investigate producer-recorded colostrum feeding practices also has limitations for the usability of the data and potential bias. The method by which first colostrum was fed was not included in our model building as we did not have confidence in how producers interpreted the question. Seventy-six percent of calves in our data were reported to have been pail-fed their first colostrum feeding, which contradicts the recommended use of a bottle or esophageal tube feeding in the North American industry (Godden et al., 2019; National Farm Animal Care Council, 2023). Due to the large discrepancy in our data, we were concerned that producers may have read the question as milk feeding, not colostrum feeding. Therefore, the feeding method variable was removed from the model building. Selection bias, mainly the Hawthorne effect, is also a limitation to be aware of. The Hawthorne effect “implies that the results are due to suggestion rather than objective environmental changes [or observations]” (Sommer, 1968). Since the producers recorded a majority of management data, some could have been more inclined to record the recommended practices from veterinarians or the Code of Practice (National Farm Animal Care Council, 2023) than the actual result, such as reporting earlier feeding times for calves. With CFT being the predominant colostrum variable, further investigation was made to ensure proper interpretation of the results. For reporting of CFT, producers answered an open-ended question for each calf about when they were fed the first feeding of colostrum after birth, and we received a range from 0.2 to 11 h. The eight farms recorded most observations within the first four hours of life (91%), with 20% of observations recorded at 1 h and 26% at 2 h of life. Individually, while still right skewed as expected, six out of eight producers had a distribution of responses past four hours. Comparing across the CFT categories (<1, 1-2, > 2 h), there was an even distribution of animals in each category and a 50% division of producers who recorded feeding a majority of calves early (<1 h) and a majority of calves late (>2 h). These results are similar to surveys conducted in the United States and Canada, where 43.6% of United States producers reported feeding calves colostrum by 2 h and 51% between 2 and 6 h (Kehoe et al., 2007), and 94.8% of Canadian producers reported feeding colostrum within the first 6 h (Vasseur et al., 2010). It is also important to consider that the time at which calves were born may be an approximation rather than a precise time. While this is an observational study on commercial farms, 24 h surveillance was not available. Calving occurring during night hours is typically unmonitored on farms; thus, the time of birth is estimated based on the wetness of the calves and their posture (lying or standing), especially when born overnight and found in the morning (Palombi et al., 2013; Manosalva et al., 2022). In our final dataset of 220 calves, only 22% were reported to be born between 2200 and 0400 hours. While the timing of overnight calving may not be as precise as those during daytime or in a controlled study design, the experience of the producers would result in a close approximation of time at birth. However, it is important also to consider the potential bias and subjectivity in producer reports.

Alternatively, FTPI status could be used instead of colostrum feeding practices. However, FTPI was not significant in our multivariable model. The use of direct and indirect measuring of IgG in serum is widespread across the literature, and it is seen as the outcome of colostrum management practices, as the presence of FTPI is directly influenced by colostrum management (Robison et al., 1988; Trotz-Williams et al., 2008; Beam et al., 2009). However, a limitation of including FTPI status in the current study was that blood samples were collected on calves between 1 and 18 d of age. Current recommendations suggest collection within the first 2 to 3 d of life and can be reliably tested up to 9 d (Wilm et al., 2018). The older a calf is, the more variable the results are and the less reliable it is in determining FTPI status (Nocek et al., 1984; Wilm et al., 2018). In the current study, only 61% of calves had blood samples collected ≤ 9 d of age. Failure of transfer of passive immunity status was still analyzed, as it was significant in univariable analysis (*P *< 0.05); therefore, FTPI status qualified for multivariable modelling (*P *< 0.2) across all production outcomes for both lactation groups. However, once included in multivariable models, FTPI was removed from backward selection or not significant (*P *> 0.05) when forced into models with and without colostrum feeding practices. The cut-off for serum IgG concentration was also addressed, as the cut-off point for FTPI has been questioned, and higher cut-offs have been proposed in recent literature ranging from 15 to 25 g/L (Chigerwe et al., 2015; Lora et al., 2019). The recommandations of Lombard et al. (2020) were considered in addition to the more traditional dichotomous cut-off of 10 g/L. Using the Lombard et al. (2020) recommendations, 62% were classified as having poor to fair IgG levels (<17.9 g/L). Using the dichotomous cut-offs of <10 and <15 g/L changed the proportion of calves with low IgG from 26% to 49%, respectively. However, the FTPI variable was not retained in the final multivariable models regardless of how we categorized the data. Even though further lab testing was not applicable for this study’s purposes, there is a need to explore the further implications of colostrum management practices on cow health, productivity, and longevity. As improvements to management can provide easy and cost-effective solutions to producers when on-farm or lab testing may not always be feasible.

Weaning weight had a significant positive trend across all production outcomes. In L1, for every 1-kg increase in WWT, production would increase by 25.5, 0.82, and 1.01 kg for milk, protein, and fat, respectively. This linear trend implies that greater WWT increases production in the first lactation, which is consistent with other studies for both WWT and preweaning average daily gain (Shamay et al., 2005; Moallem et al., 2010; Soberon et al., 2012). Conversely, several experiments have reported adverse long-term effects from increased WWT, especially from pre-pubertal compensatory growth, as studies have found reduced development of mammary tissue and increased fat pad size, leading to reduced milk yield (Sejrsen et al., 2000; Thorn et al., 2008; Soberon et al., 2012). Similar effects are seen in genetic selection, where body weight and conformation have shown strong positive correlations with an animal’s milking ability but also have strong negative genetic correlations with fertility and health traits (Miglior et al., 2017). While adverse effects of WWT were not observed in this study, it is an important consideration for future studies focusing on WWT from a genetic or environmental context. It is also important to note that WWT can be heavily influenced by weaning age and feeding and management protocols (Heinrichs and Heinrichs, 2011). However, WWT was our study’s only consistently recorded variable, as some animals were missing a weaning date. Assessment of WWT has also been shown to have improvements on the age at first calving and production (Chuck et al., 2018); thus, including WWT in analysis was still deemed a viable option.

The occurrence of disease and antibiotic treatment during the preweaning period was negatively associated with L1 fat yields. While disease was the only significant association, there was a significant interaction between disease and antibiotic treatment. Overall, healthy and untreated calves produced the 305 d greatest fat yield, compared to calves that were diseased-treated, healthy-treated, and diseased-untreated in that order. These negative associations are most likely due to sickness-related factors (decreased feed intake, lethargy, malaise), which divert nutrients to healing instead of growth (Johnson, 1998; Dantzer, 2009). For animals that were healthy and treated with antibiotics, it is suspected that some producers may have been treating a larger population of calves at once to stop disease transmission, or they failed to document the disease for which they administered antibiotics in their records. Heinrichs and Heinrichs (2011) reported a negative effect of illness on first lactation fat production, which is consistent with our findings. However, they reported a much lower overall impact of disease on 305 d fat yield of −5.18 kg (S.E: 1.78 kg) compared to our study of −80.5 kg (S.E: 28.9kg).

Soberon et al. (2012) also reported a negative association with adult milk production when antibiotics were administered during the preweaning period; however, no estimates regarding fat yield specifically were reported. The difference in estimates may be due to our study’s low disease prevalence. Out of 220 calves, only 10% had a reported morbidity consisting of calf diarrhea (5.45%), respiratory disease (2.27%), and navel infection (2.27%). Comparatively, in the 2014 U.S. national survey, morbidity was much greater at 33.8%, with 50.9% attributed to calf diarrhea and 28.1% to respiratory disease (NAHMS, 2021a). The low prevalence observed in our study was potentially due to underreporting by producers, as there were no standardized protocols for identifying disease in our study or nationally. While the producer-reported cases of disease were reviewed by the herd veterinarian shortly after, producers may have been inclined only to record disease or inform the veterinarian in more severe circumstances that required treatment compared to cases that would have resolved naturally. In addition, the participating herds may not be representative of a larger population, as it was voluntary participation, and healthier herds may have been more inclined to participate. Even with potential underreporting, the trends of a) healthy calves producing greater outputs compared to those who are unhealthy and b) those treated with antibiotics are likely to have a better outcome than their unhealthy, untreated counterparts, are still biologically relevant in our analysis. The difference between studies may also be attributed to our inclusion of GPA, which impacted the magnitude of our results. Future research involving disease incidence would benefit by utilizing a standardized scoring system for different calf diseases, such as those proposed by McGuirk and Peek (2014). A more consistent definition of disease would allow for more accurate and relevant comparisons between studies in the future (van Staaveren et al., 2023) and more accurate reporting of disease to help inform management decisions on farm.

The models for L2 + 3 were the same across all production outcomes, where a positive interaction effect was present between CFT and WWT. The reasoning for investigating a CFT and WWT interaction was possible long-term health issues due to an inadequate transfer of passive immunity (Fischer et al., 2018), resulting in decreased weaning weights, which, as mentioned previously, has shown to decrease production as an adult (Soberon et al., 2012; Volkmann et al., 2019). Due to the biology of the variables, the interaction will always be present because a calf will always have a CFT and WWT (no null effect present). We found that increased WWT coupled with later CFT groups of 1-2 h and > 2 h produced greater production outputs in L2 + 3 when WWT was above the median of 84.5 kg. These trends were comparable to previous studies, where increased WWT and CFT under 6 h were associated with greater production outputs (Moallem et al., 2010; Fischer et al., 2018; Armengol and Fraile, 2020). The general recommendation for colostrum feeding is as soon as possible, or at least within 4 to 6 h of birth (National Farm Animal Care Council, 2023). In our study, we still see a positive effect of CFT > 2 h with WWT. The positive interaction we see is most likely due to the data distribution in the CFT > 2 h group, as 75% are fed between 2 and 4 h, while the remaining 25% are spread out from 4.5 to 11 h. In addition, Shamay et al. (2005) raised the possibility that nursing management in association with increased growth could be instrumental in triggering earlier puberty onset, which is commonly associated with increased milk yields. In the present study, it seems this association may be an underlying factor for why CFT and WWT present an effect together.

There is an increasing body of evidence supporting the various impacts that preweaning calf management and disease have on first lactations. Most focus on colostrum factors, average daily gain, and disease occurrence. However, limited literature reports calf management impacts past the first lactation (Heinrichs and Heinrichs, 2011; Soberon et al., 2012; Curtis et al., 2018). The limiting factor is likely due to the time and resources required to attempt studies past the first lactation. While this study was able to estimate some of the effects of preweaning management on first lactation in addition to the second and third lactation, it is important to note the limitations due to the voluntary participation and retrospective nature of this study. First, due to the number of participating herds, the information may not represent a larger population past New Brunswick, Canada. While our study may have information on more herds than other studies to account for farm-dependent factors (Aghakeshmiri et al., 2017; Abuelo et al., 2021), participation restricted to a smaller geographic location is a limiting factor. It is also important to be aware of volunteer participation in our study as these herds may not represent certain management practices. Volunteers may be more inclined to participate if they believe they have greater production or good management operations, which may skew the results. As with most retrospective studies, the data were collected originally for other purposes. Therefore, some data that would have been ideal for our objective was not collected, or measurement could have been conducted differently. For example, more training and validation for individual calf logs to ensure the limited presence of the Hawthorne effect and testing for FTPI within the first 7 d of life. It is essential to assess the implications of preweaning management practices that could impact the production and longevity of calves with a prospective study.

Future studies should attempt to collect detailed calf health and management data regularly with standardized protocols, allowing for more detailed investigations on the impact of calf management practices on later lactations. Adding a genomic calf evaluation for Canadian selection indexes would also help promote regular recording on farms and further evaluate the impact of calf genetics. By investigating the genetic potential, preweaning colostrum practices, management factors, and health measures together, we can more accurately identify areas to optimize management, which can improve production. The findings in this study are helpful to begin conversations with producers and veterinarians regarding the importance of calf management not only on calf health but also the production of a dairy cow when they are in the milking herd.

## Conclusion

The CFT and WWT impacted adult milk and protein yield production in L1 and L2 + 3, and fat yield production in L2 + 3, with CFT and greater WWT correlating with greater production yields. Disease and antibiotic treatment negatively impacted fat yield in the first lactation. All models were improved with the addition of GPA and provided increased accuracy of estimations from this study. These results help to corroborate that colostrum management, WWT, and diseases in the preweaning period can impact adult production while accounting for an animal’s genetic potential in herds within New Brunswick, Canada.

## Supplementary Material

skae061_suppl_Supplementary_Material

## Abbreviations

CFT: colostrum feeding time
FTPI: failure of transfer of passive immunity
GEBV: genomic estimated breeding value
GPA: genomic parent average
IgG: immunoglobulin G
L1: lactation 1
L2 + 3: lactations 2 and 3
NBDGP: New Brunswick Dairy Genome Project
WWT: weaning weight
