# Supplementary material for: Impacts of preweaning colostrum feeding practices and health measures on dairy cow production, while accounting for genetic potential
Source: J Anim Sci. 2024 Mar 9;102:skae061. doi: 10.1093/jas/skae061 (PMC10998465; doi:10.1093/jas/skae061)
Supplement: skae061_suppl_Supplementary_Material [file skae061_suppl_supplementary_material.docx]

**SUPPLEMENTARY MATERIAL**

The final statistical models are presented below in symbolic notation. Categorical variables are denoted by α, and quantitative variables are denoted by β.

The models for L1 production are presented below:

The models for L2+3 production are presented below:

where the outcome variables were milk yield (MY), fat yield (FY), and protein yield (PY), for respective lactation group designated by the subscript. Predictive variables included in the model were: H = herd, CFT = first colostral feeding time after birth (0 = ≤1 h; 1 = 1 – 2 h; 2 = >2 h), WWT = weaning weight of calf (kg), D = diseased with scours, pneumonia, or navel infection (1 = healthy; 2 = diseased), TX = treatment with antimicrobials (1 = no treatment; 2 = treatment), GPA_M_ = genomic parent average for the milk yield, GPA_F_ = genomic parent average for fat yield, GPA_P_ = genomic parent average for protein yield, L = lactation number, and the random effects were as follows: a = cow-level cluster effects, e = residual.
